# miR‐30b Promotes spinal cord sensory function recovery via the Sema3A/NRP‐1/PlexinA1/RhoA/ROCK Pathway

**DOI:** 10.1111/jcmm.15591

**Published:** 2020-09-25

**Authors:** Xin Wang, Bo Li, Zhijie Wang, Fengyan Wang, Jing Liang, Chuanjie Chen, Lei Zhao, Bo Zhou, Xiaoling Guo, Liqun Ren, Xin Yuan, Xueming Chen, Tianyi Wang

**Affiliations:** ^1^ Chengde Medical University Chengde China; ^2^ Department of Orthopedics Sun Yat‐Sen Memorial Hospital of Sun Yat‐Sen University Guangzhou China; ^3^ Department of Pediatric Internal Medicine Affiliated Hospital of Chengde Medical University Chengde China; ^4^ Department of Orthopedics 981st Hospital of the Chinese People's Liberation Army Joint Logistics Support Force Chengde China; ^5^ Department of Nursing 981st Hospital of the Chinese People's Liberation Army Joint Logistics Support Force Chengde China; ^6^ Department of Orthopedics Chengde Central Hospital Chengde China; ^7^ Department of Education Affiliated Hospital of Chengde Medical University Chengde China; ^8^ Department of Neurology 981st Hospital of the Chinese People's Liberation Army Joint Logistics Support Force Chengde China; ^9^ Laboratory of Spinal Cord Injury and Rehabilitation Chengde Medical University Chengde China; ^10^ Department of Spine Surgery, Beijing Luhe Hospital Capital Medical University Beijing China

**Keywords:** miR‐30b, primary sensory neuron, RhoA, sema3A, spinal cord injury

## Abstract

Spinal cord injury (SCI) induces both motor and sensory dysfunctions. We wondered whether miR‐30b could promote primary sensory neuron (PSN) axon growth in inhibitory microenvironment. The neurite growth was promoted by miR‐30b agomir and inhibited by antagomir. MiR‐30b targeted and degraded sema3A mRNA. MiR‐30b regulated the formation of sema3A‐NRP‐1‐PlexinA1 complex via targeting sema3A. The neurite length was induced by the miR‐30b agomir, and the application of sema3A protein could reverse the effect of agomir. GTP‐RhoA and ROCK expression were down‐regulated by miR‐30b. Neurite outgrowth that inhibited by sema3A and the miR‐30b antagomir was increased by Y‐27632. Agomir promoted neurite growth in NogoA inhibitory conditions, which indicated miR‐30b could both enhance neuronal intrinsic regenerative ability and promote neurite growth against inhibitory microenvironment via Sema3A/NRP‐1/PlexinA1/RhoA/ROCK axis. The agomir could also regulate Sema3A/NRP‐1/PlexinA1/RhoA/ROCK axis in vivo and restore spinal cord sensory conductive function. In conclusion, miR‐30b could be a novel target for sensation recovery after SCI.

## 1 INTRODUCTION

Spinal cord injury (SCI) is a crippling disease that places a heavy burden on both patients and the society due to the loss of motor and sensory function and the high cost of treatment and rehabilitation.^1^, ^2^, ^3^ To date, the underlying mechanism of SCI remains unclear, which directly limits the development of effective clinical treatment strategies. Different from locomotor dysfunction that prevents patients from moving their body, sensory dysfunction exerts a more serious effect on patients’ daily life. Sensory function disorders, including sensory loss, neuralgia, dysesthesia and paraesthesia, torture patients incessantly.^4^ Unfortunately, sensory disorders are difficult to handle in the clinic due to limited effective treatment strategies. Central neuropathic pain induced by SCI is insensitive to pharmacological, surgical, physical, and behavioural treatments.^5^ Thus, developing efficient treatment strategies to promote the recovery of sensory function is of particular importance to SCI patients.

Both motor and sensory conduction functions are interrupted because the axons are ruptured by primary traumatic injury and axon degradation in the secondary phase of SCI. The recovery of both motor and sensory functions relies on the reconnection of the interrupted axons. Facilitating neuronal axon regeneration is the most important process to restore the function of injured spinal cord. However, the regeneration of neuronal axons is determined by both the neuronal intrinsic regenerative ability and extrinsic axon growth microenvironment, which is composed of both neurotrophins and inhibitory molecules. Studies focusing on a strategy of neutralizing the inhibitory molecules have shown a limited effect on axon regeneration. Therefore, aiming to enhance the neuronal intrinsic regenerative capability after injury is of great importance.^6^ Semaphorin‐3A (Sema3A), a neuronal secreted chemorepulsive guidance cue that binds to the PlexinA1 and NRP‐1 co‐receptor, induces neuronal growth cone collapse in an autocrine manner and modulates RhoA/ROCK pathway in PC12 cells and rat Parkinson disease model.^7^, ^8^, ^9^, ^10^ Thus, sema3A can regulate neuronal axon growth via both intrinsic and extrinsic manner. RhoA/ROCK pathway is recognized as a negative modulatory pathway that inhibits neuronal axon growth. Axon growth inhibitory factors, such as NogoA, exert their negative regulatory function via RhoA/ROCK pathway.^11^ RhoA/ROCK pathway is reported to be downstream of sema3A.^12^ Therefore, inhibiting the Sema3A/NRP‐1/PlexinA1/RhoA/ROCK pathway could be a novel treatment strategy to induce primary sensory neuronal axon growth post‐SCI.

**Figure 1:**
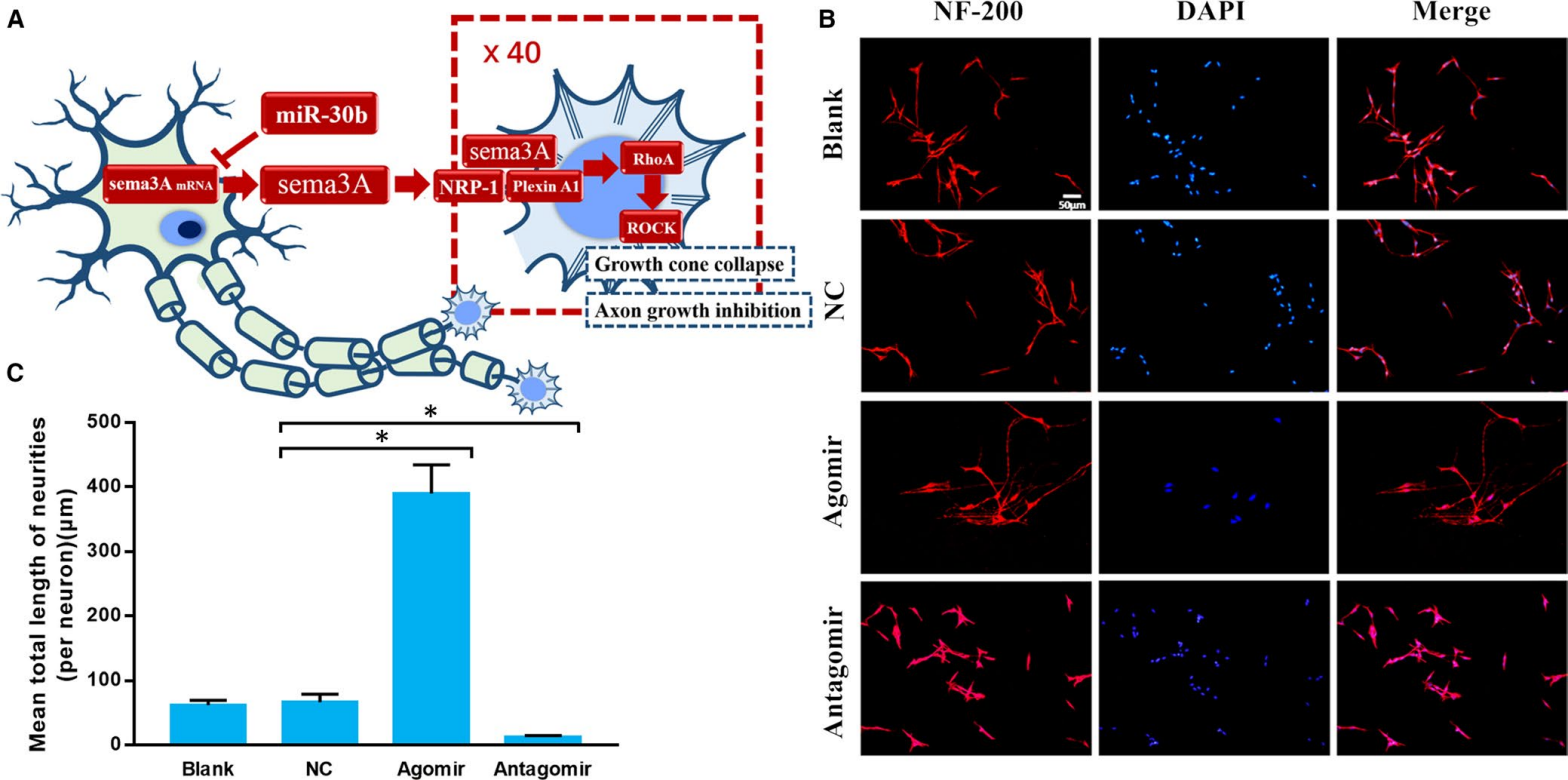
miR‐30b promotes primary sensory neuron axon growth in vitro. A, Schema diagram of the hypothesis. B, Immunocytofluorescence images of cultured primary sensory neurons. Neurons are red, and cell nuclei are blue (200×). C, The mean total neurite length (per neuron). The data are expressed as the mean ± SD (one‐way ANOVA followed by Tukey's test; n = 4; *P < .05). (Blank: normal culture condition with no treatment; NC: negative control, treated with scramble sequence)

MicroRNAs (miRNAs) are a cluster of highly conserved RNAs that modulate various genes expression by binding to the mRNA 3’UTR of target gene to induce mRNA degradation or translation inhibition.^13^, ^14^, ^15^, ^16^ Meanwhile, they are widely expressed in both the central nerve system and peripheral nerve system and have been shown to be involved in regulation of neuronal axon growth ability.^17^, ^18^ In Parkinson's disease, miR‐30b shows cytoprotective role via targeting SNCA and inhibiting cell apoptosis.^19^ MiR‐30b overexpression relieves neuropathic pain.^20^, ^21^, ^22^ MiR‐30b has been shown to target sema3A to promote retinal ganglion cell neurite growth.^23^ These evidences indicate that miR‐30b exerts beneficial effect in central nerve system. However, there is no study on the role of miR‐30b in spinal cord injury. Based on similarities in structure and function among different kinds of neurons and database prediction, we speculated that miR‐30b could be involved in the process of regulating primary sensory neuronal axon growth via targeting sema3A in spinal cord dorsal column lesion (SDCL) and try to explore its potential mechanism. Simultaneously, we wondered whether RhoA/ROCK pathway regulated by sema3A participates in primary sensory neuron axon growth. We sought to clarify whether miR‐30b could enhance neuronal intrinsic regenerative abilities and promote neurite growth against inhibitory microenvironment via Sema3A/NRP‐1/PlexinA1/RhoA/ROCK axis. A schema diagram of this hypothesis was presented in Figure 1A. In vivo experiment, we regulated miR‐30b expression in dorsal root ganglion (DRG) to verify the repair effect of miR‐30b/Sema3A/NRP‐1/PlexinA1/RhoA/ROCK axis on the injured dorsal column.

In this study, we conducted in vitro study to verify the role of miR‐30b on primary sensory neuron neurite outgrowth and the expression of target gene and downstream genes. Further, the treatment effect of miR‐30b on sensory function and the regeneration of neural axons that located in spinal cord dorsal column was detected in SDCL rats. To our knowledge, this was the first study that demonstrated miR‐30b regulates primary sensory neuron axon growth and spinal cord sensory conductive function recovery via the Sema3A/NRP‐1/PlexinA1/RhoA/ROCK pathway. This study clarified the new molecular mechanism of SCI and provided a new potential treatment target to restore sensory conductive function post‐SCI.

## 2 MATERIALS AND METHODS

### 2.1 Ethics statements

40 adult, female Wistar rats (200 g ± 50 g) were bought from the Animal Center of Tianjin Radiation Study Institute. 4 rats were applied to obtain primary sensory neurons (DRG neurons), and 36 rats were used for in vivo experiments. All the required procedures were performed according to the ARRIVE guidelines (www.nc3rs.org.uk/ARRIVE) and National Institutes of Health Guide for the Care and Use of Laboratory Animals (NIH Publication no. 85‐23, revised 1996). This study was approved by the Ethics Committee of the 981st Hospital of the Chinese People's Liberation Army Joint Logistics Support Force (Approval No. 20170038).

### 2.2 Primary sensory neuron culture in vitro

Primary sensory neurons were cultured in vitro according to a protocol described previously.^16^ Briefly, the Wistar rats were anaesthetized with chloral hydrate (10%, 3.3 mL/1 kg) and sterilized with 75% alcohol. Then, the DRG tissues were extracted and cut into pieces in cold DMEM/HG (Gibco, Waltham, MA, USA). The DRG tissues were incubated with 0.125% trypsin (Gibco, Waltham, MA, USA) in DMEM/HG for 30 minutes. After centrifugation (5 minutes, 1000 rpm/min), the cells were suspended in Neurobasal Medium (Invitrogen, Carlsbad, USA). The culture medium was supplemented with NGF (50 ng/mL, Sigma‐Aldrich, St. Louis, USA), B‐27 (20 μL/mL, Invitrogen, Carlsbad, USA) and L‐glutamine (1%, 0.2 mol/L, Gibco, USA). The cells were seeded into poly‐L‐lysine coated plates. The next day, NogoA‐Fc (4 mg/mL, R&D Systems, Minneapolis, USA) was used to mimic the inhibitory environment in vitro. Then, the miR‐30b agomir/antagomir or negative control and sema3A siRNA were added for transfection. The sema3A (100 ng/mL, R&D Systems, Minneapolis, USA)^24^ and Y‐27632 (10 μmol/L, Selleckchem, Munich, Germany) were added into the culture medium immediately after transfection.

### 2.3 Transfection

To clarify the role of miR‐30b in cultured DRG neurons, the neurons were transfected with miR‐30b agomir/antagomir or negative control (100 pmol miR‐30b agomir: 5′‐UGUAAACAUCCUACACUCAGCU‐3′ and 3′‐CUGAGUGUAGGAUGUUUACAUU‐5′; 100 pmol miR‐30b antagomir: 5′‐AGCUGAGUGUAGGAUGUUUACA‐3′; 100 pmol negative control: 5′‐UUCUCCGAACGUGUCACGUTT‐3′ and 3′‐ACGUGACACGUUCGGAGAATT‐5′) and sema3A siRNA (5′‐GCAAUGGAGCUUUCUACUA‐dTdT‐3′) (GenePharma, Shanghai, China) with Lipofectamine 2000 (Invitrogen, Carlsbad, CA). 24 hours after transfection, the culture medium was removed and fresh complete Neurobasal Medium (Invitrogen, Carlsbad, USA) was added. Then, the DRG neurons were harvested for RT‐qPCR, Western blotting and immunocytofluorescence 48 hours after transfection.

### 2.4 Luciferase assay

The 3’UTR fragment of sema3A was amplified and subcloned into a pMIR‐REPORTTM luciferase reporter vector (Ambion, Austin, TX, USA) to generate sema3A‐WT. Using the same process, a sema3A‐MUT pMIR‐REPORTTM luciferase reporter vector containing a mutant sequence at the predicted miR‐30b binding sites was constructed. HEK293T cells were cotransfected with the reconstructed reporter vectors (sema3A‐WT or sema3A‐MUT, 0.5 μg/well) and miR‐30b or miR‐NC (100 pmol). Luciferase activity was detected 24 hours post‐transfection with a Dual‐Glo Luciferase Reporter Assay (Promega, USA, Madison).

### 2.5 Spinal cord dorsal column lesion (SDCL)

To clarify the treatment effect of miR‐30b agomir in vivo, 36 rats were separated randomly into Sham, Scramble (SDCL + scramble) and Agomir (SDCL + miR‐30b agomir) groups (12 rats per group). The SDCL model was built as previously reported.^25^ Briefly, the spinal cord was exposed after T10 laminectomy. The dorsal tissue of spinal cord between two dorsal roots of DRGs was crushed with fine forceps 2 mm in depth. At the same time, the L4‐6 DRG tissues were exposed. Agomir solution was injected into L4‐6 DRG tissues with micro‐injection pump as our previous study described (400 μm in depth, 0.2 μL/min, 1.1μl solution).^16^ Then, the incisions were sutured and the rats were sent back to cages.

### 2.6 Reverse transcription quantitative real‐time polymerase chain reaction (RT‐qPCR)

To detect the expression of sema3A mRNA in cultured DRG neurons after miR‐30b modulation, RT‐qPCR was performed according to a protocol described previously.^16^, ^26^ Cultured DRG neurons were harvested, and the cells were lysed with a TRIzol kit (Invitrogen, Carlsbad, USA) to extract the total RNA. Then, cDNA was synthesized with a cDNA Reverse Transcription Kit (Thermo Fisher Scientific, Waltham, MA, USA). miScript SYBR® Green PCR Kit (QIAGEN, Dusseldorf, Germany) was used to detect the expression of sema3A mRNA. GAPDH mRNA was chosen as an internal reference. RT‐qPCR was conducted on a LightCycler® 480 II RT‐PCR instrument (Roche Diagnostics, Basel, Switzerland). The samples were detected at least three times.

### 2.7 Western blotting and Immunoprecipitation

Protein expression was detected via Western blotting as reported previously.^16^ DRG neurons were harvested, and the proteins were extracted with RIPA buffer (Santa Cruz Biotechnology, Inc, Dallas, TX, USA). A protease inhibitor cocktail (Sigma‑Aldrich, St. Louis, MO, USA) was used to inhibit the degradation of proteins. After determining the protein concentration of samples with a Nandrop2000 spectrophotometer (Thermo Scientific, Waltham, MA, USA), the proteins were separated with SDS‐PAGE. Then, the proteins were transferred to a PVDF membrane. The primary antibodies (anti‐sema3A, anti‐PlexinA1, anti‐NRP‐1, anti‐RhoA, anti‐ROCK and anti‐actin) and horseradish peroxidase‐conjugated secondary antibody (Abcam, Cambridge, UK) were applied to detect the expression of the indicated proteins. For immunoprecipitation, sema3A antibody was incubated with samples (4 ℃/ overnight). Then, the indicated complexes were precipitated and detected using Western blotting. The samples were detected at least three times.

### 2.8 Assessment of primary sensory neuron neurite length

The cultured DRG neurons were fixed with 4% paraformaldehyde. After blocking, the neurites were probed with anti‐NF‐200 antibody (1:200; Abcam, Cambridge, UK) and anti‐IgG‐Cy3 (1:200, Abcam, Cambridge, UK). The nuclei were stained with DAPI (D9564, Sigma). The mean total neurite length was calculated from one hundred neurons per well with Image‐Pro Plus (IPP) 6.0 software (Media Cybernetics, Silver Spring, USA).

### 2.9 Target gene prediction

We utilized four databases, MIRNAMAP, TARGETSCAN, STARBASE AND MIRWALK2 (mirnamap.mbc.nctu.edu.tw/index.php, www.targetscan.org/vert_72/, starbase.sysu.edu.cn/index.php, and zmf.umm.uni‐heidelberg.de/apps/zmf/mirwalk2/index.html), to predict the potential miRNAs that target sema3A. The common miRNAs predicted by the four databases were calculated with the Bioinformatics & Evolutionary Genomics website (bioinformatics.psb.ugent.be/webtools/Venn/).

### 2.10 Immunofluorescence

The NF‐200^+^ axons were probed with immunofluorescence staining. Briefly, the spinal cord (8 weeks after injury) was harvested and sliced horizontally (16 μm). The slices were probed with primary antibody (NF‐200, 1:200; Abcam) and secondary antibody (goat anti‐rabbit IgG‐cy3, 1:200; Abcam). The pictures were taken and analysed with Image‐Pro Plus 6.0 (Media Cybernetics, Silver Spring, MD, USA).

### 2.11 Somatosensory evoked potentials (SSEP)

The spinal cord sensory conductive function was detected by SSEP as our previous study reported.^25^ Briefly, the detection system was connected to rats with electronic needles. The stimulator electrode needles were inserted into the muscle near the sciatic nerve, and the recording needle was placed subcutaneously in the head. Stimulation strength (2.5 mA, 4.1 Hz) was set, and the SSEP waveform was recorded and averaged from 200 stimulation. The N peak latency and N‐P peak amplitude were recorded and analysed to reflect the spinal cord sensory conductive function.

### 2.12 Tape remove test (TRT)

Tape remove test is a sensitive test to detect the sensory function after spinal cord injury.^27^ Briefly, a tape was adhered to the hindlimbs, and the latency of sensing and removing the tape was recorded. The TRT was conducted at −1 w, −1 d, 1 w, 2 w, 3 w, 4 w, 5 w, 6 w, 7 w and 8 w timepoints. If the latency was longer than 120s, we recorded 120s as the latency time.

### 2.13 Statistical analysis

The data were shown as the mean ± standard deviation (SD). One‐way ANOVA with Tukey's multiple comparison test was applied to analyse differences. *P*‐values < .05 were considered statistically significant. All tests were performed at least three times.

## 3 RESULTS

### 3.1 miR‐30b promotes primary sensory neuron axon growth in vitro

To clarify the modulatory role of miR‐30b in axon outgrowth, cultured primary sensory neurons were transfected with miR‐30b agomir, antagomir or negative control. The mean total neurite length of cells in the blank, negative control, agomir and antagomir groups was calculated manually. The agomir group showed the longest neurites, while antagomir significantly inhibited neurite growth compared with that in the NC group (Figure 1B,C). These data indicated that miR‐30b could promote primary sensory neuron axon growth, but the underlying mechanism was still ambiguous.

### 3.2 miR‐30b targets sema3A

**Figure 2:**
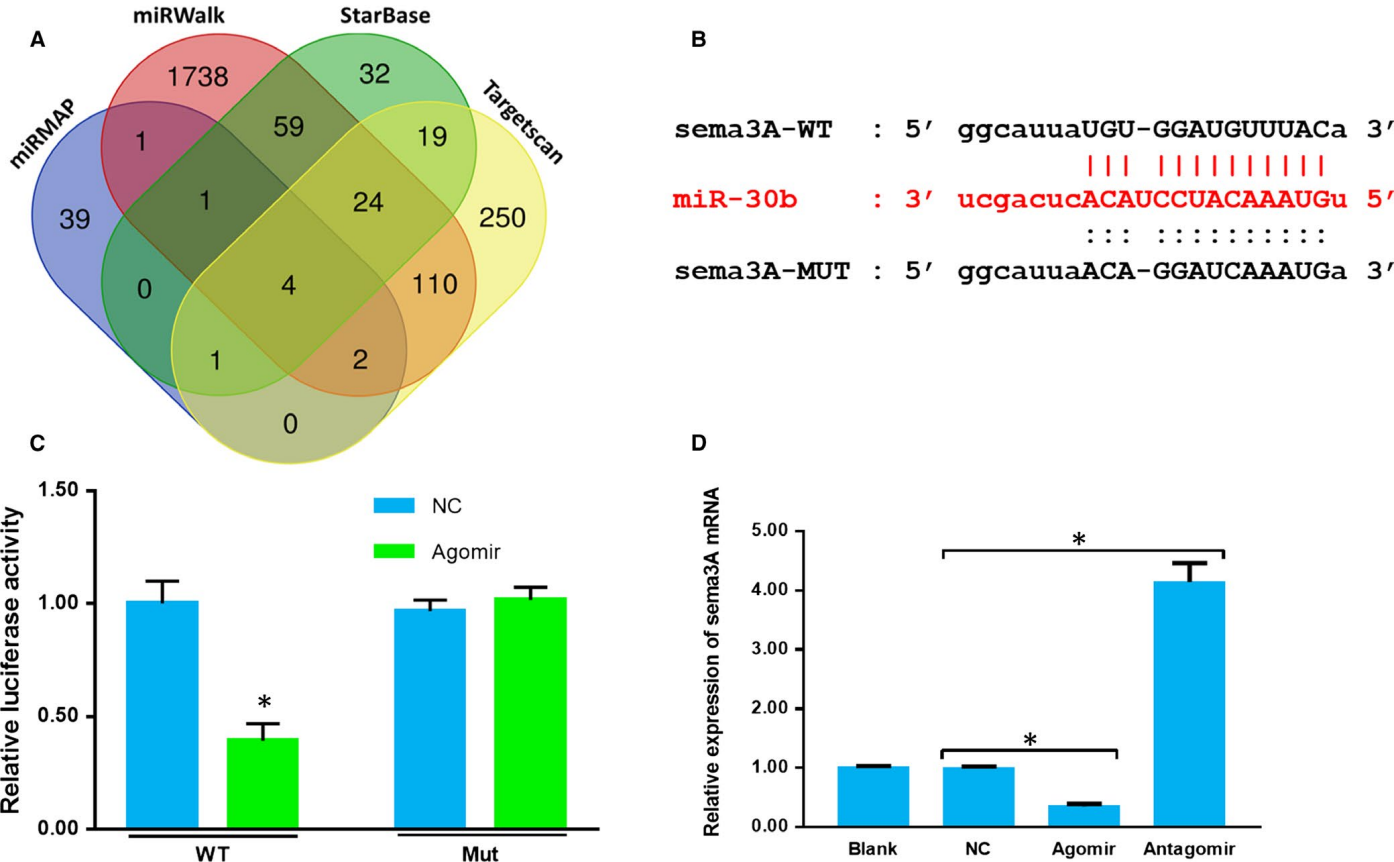
miR‐30b targets sema3A. A, Venn diagram showing the common miRNAs predicted by four databases. B, The predicted binding and mutant sites of miR‐30b and sema3A mRNA. C, Dual‐luciferase assay. D, The relative expression of sema3A mRNA. The data are expressed as the mean ± SD (WT: wild type; Mut: mutant. One‐way ANOVA followed by Tukey's test; n = 4; *P < .05)

Bioinformatic prediction predicted these miRNAs that potentially targeted sema3A. The miRNAs predicted by at least three databases were shown in Figure 2A and Table 1. MiR‐30b‐5p (miR‐30b), miR‐30c‐5p, miR‐30d‐5p and miR‐30e‐5p were predicted by all four databases. MiR‐30b‐5p has been proven to target sema3A in retina ganglion cells.^23^ Thus, we presumed that miR‐30b might target sema3A in primary sensory neurons. The potential binding sites of miR‐30b and sema3A mRNA and the sema3A mRNA 3’ UTR mutant sites for the dual‐luciferase assay were shown in Figure 2B. Next, we conducted a dual‐luciferase assay to prove the interaction of miR‐30b and sema3A mRNA (Figure 2C). The luciferase activity in the WT+ agomir group was evidently inhibited, while there were no differences in luciferase activity among the other three groups (*P* > .05; WT + miR‐NC, MUT + miR‐NC and MUT + agomir). These data indicated that miR‐30b could inhibit the expression of sema3A by interacting with sema3A mRNA 3’UTR.

**Table 1:** Predicted miRNAs from four databases

Name	Total	Elements
StarBase TargetScan miRNAMap miRWalk	4	miR‐30d‐5p, miR‐30b‐5p, miR‐30e‐5p, miR‐30c‐5p
StarBase miRNAMap miRWalk	1	miR‐448
TargetScan miRNAMap miRWalk	2	miR‐297, miR‐380‐5p
StarBase TargetScan miRNAMap	1	miR‐30a‐5p
StarBase TargetScan miRWalk	24	miR‐369‐3p, miR‐411‐5p, miR‐16‐5p, miR‐363‐3p, miR‐25‐3p, miR‐330‐3p, miR‐106b‐5p, miR‐145‐5p, miR‐497‐5p, miR‐196b‐5p, miR‐196a‐5p, miR‐19b‐3p, miR‐93‐5p, miR‐195‐5p, miR‐15b‐5p, miR‐490‐3p, miR‐153‐3p, miR‐21‐5p, miR‐493‐5p, miR‐383‐5p, miR‐92a‐3p, miR‐129‐5p, miR‐216a‐5p, miR‐155‐5p

However, it was still unclear whether miR‐30b could inhibit sema3A expression via mRNA degradation or translation inhibition. The expression of sema3A mRNA in primary sensory neurons was detected after miR‐30b agomir or antagomir transfection. Agomir significantly down‐regulated the expression of sema3A mRNA and antagomir evidently up‐regulated sema3A mRNA expression compared with that in NC group (Figure 2D) (*P* < .05). These data indicated that miR‐30b could inhibit sema3A expression by binding to the 3’UTR and inducing degradation of sema3A mRNA in primary sensory neurons.

### 3.3 miR‐30b regulates the sema3A/PlexinA1‐NRP‐1 axis

**Figure 3:**
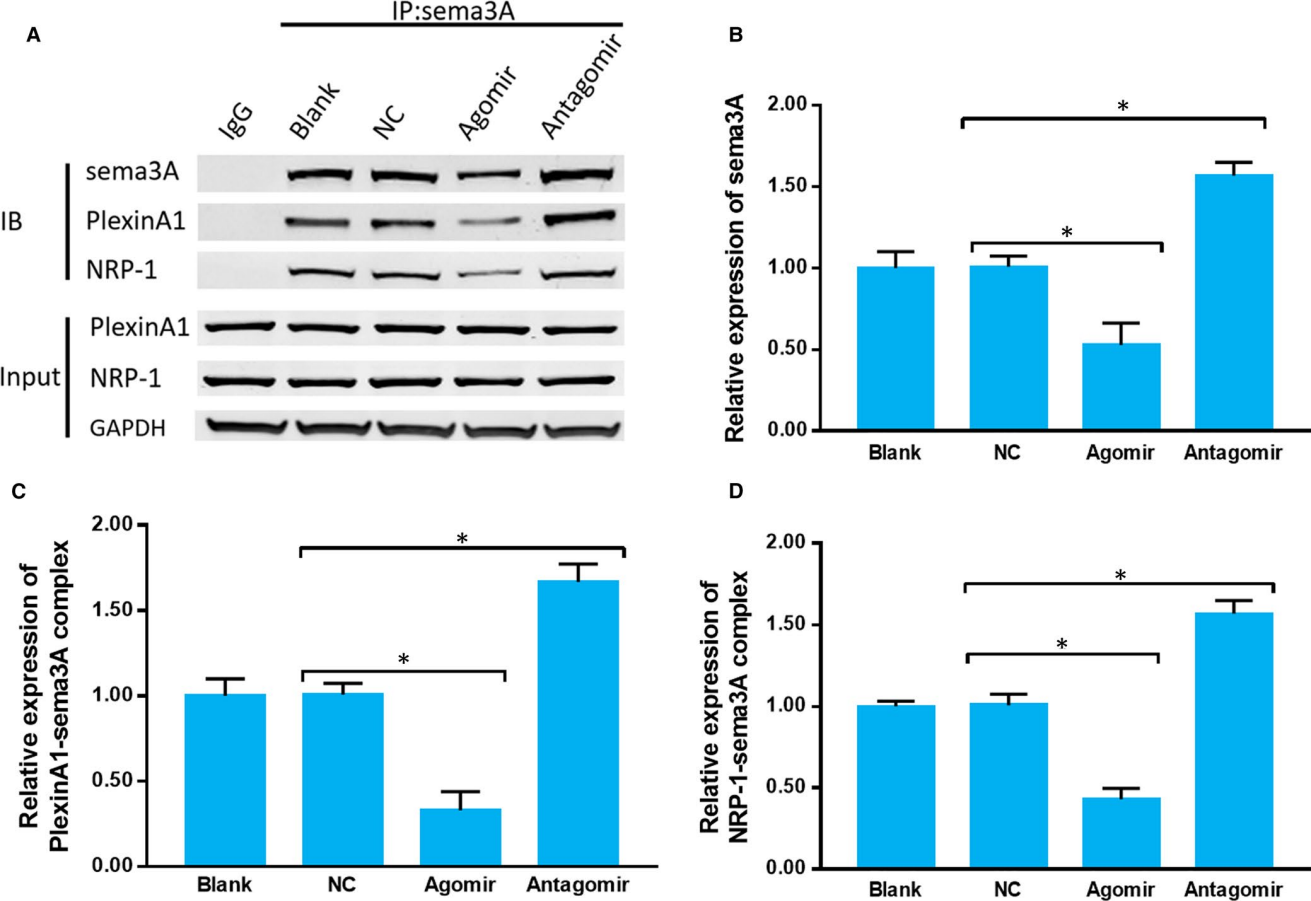
miR‐30b regulates the sema3A/PlexinA1‐NRP‐1 axis. A, The binding of sema3A, NRP‐1 and PlexinA1 under miR‐30b modulation was detected by coimmunoprecipitation assay. B‐D, Quantitative histogram showing the expression of sema3A, PlexinA1‐sema3A complex and NRP‐1‐sema3A complex. The data are expressed as the mean ± SD (one‐way ANOVA followed by Tukey's test; n = 4; *P < .05)

Previous studies have demonstrated that sema3A is a ligand that binds to the co‐receptor formed by PlexinA1 and NRP‐1 to trigger intracellular signalling.^28^, ^29^ Therefore, we detected the binding of sema3A, PlexinA1 and NRP‐1 in cultured primary sensory neurons after miR‐30b modulation. Agomir evidently decreased the expression of sema3A, and antagomir significantly up‐regulated its expression compared with their levels in NC group. The formation of sema3A‐NRP‐1 and sema3A‐PlexinA1 complexes was increased in antagomir group and decreased in agomir group compared with that in NC group (Figure 3A‐D). These data showed that miR‐30b regulates sema3A/PlexinA1‐NRP‐1 axis via targeting sema3A and modulating the formation of sema3A/PlexinA1‐NRP‐1 complex in cultured primary sensory neurons.

### 3.4 Sema3A is the key target gene of miR‐30b to regulate axon growth in primary sensory neurons

**Figure 4:**
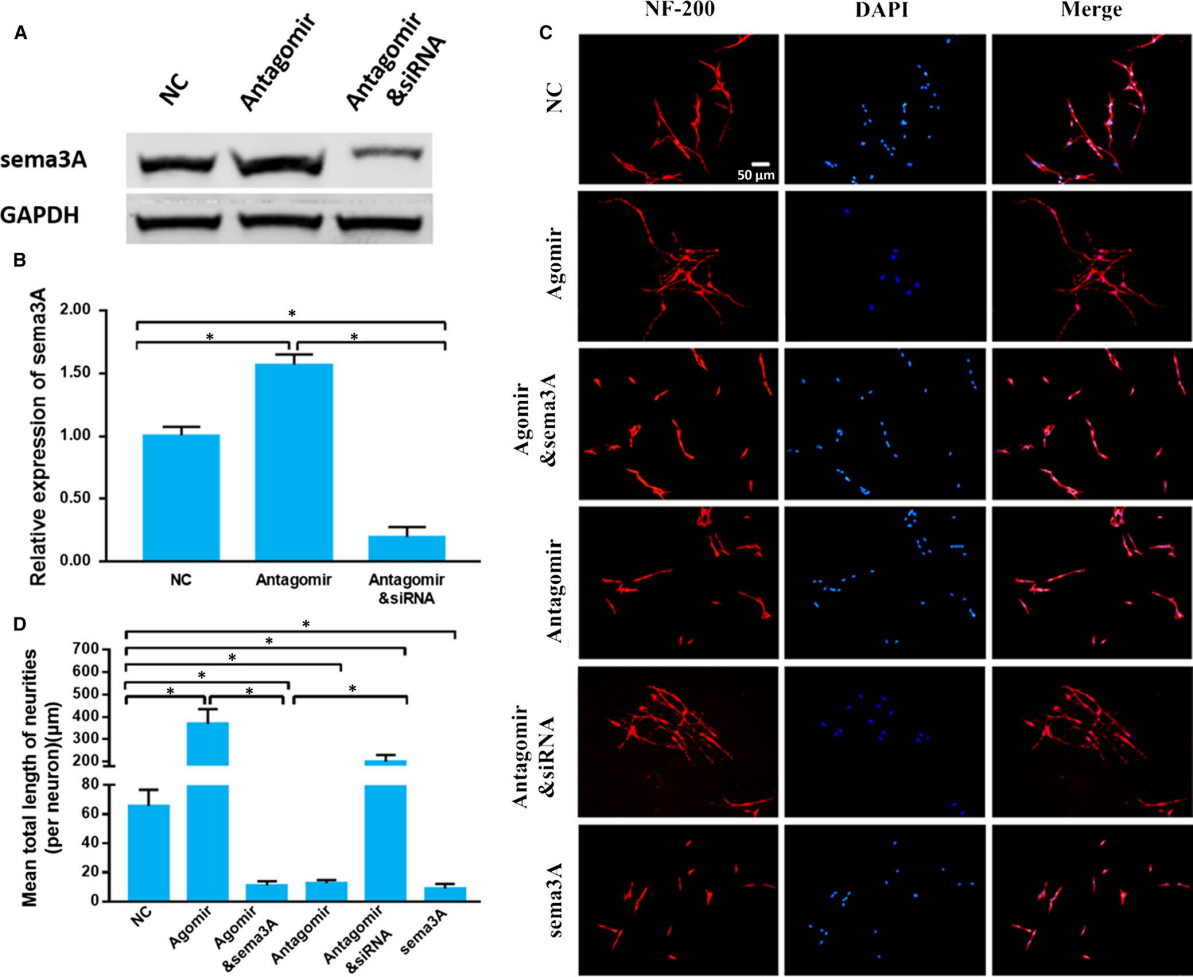
Sema3A is the key target gene of miR‐30b to regulate axon growth in primary sensory neurons. A‐B, The relative expression of sema3A detected by Western blot normalized to GAPDH expression. C, Immunocytofluorescence images of the primary sensory neurons. Neurons are red, and cell nuclei are blue (200×). D, The mean total neurite length (per neuron). The data are expressed as the mean ± SD (one‐way ANOVA followed by Tukey's test; n = 4; *P < .05)

Because one miRNA can regulate the expression of multiple genes, it is necessary to verify whether other target genes of miR‐30b could also regulate axon growth. We transfected sema3A siRNA to knock‐down sema3A expression. In antagomir group, the expression of sema3A was up‐regulated, while in antagomir plus sema3A siRNA group, the expression of sema3A was down‐regulated. These results indicated that sema3A siRNA inhibited sema3A expression (Figure 4A,B). Antagomir significantly inhibited axon growth; however, sema3A siRNA significantly blocked this inhibition (*P* < .05). We wondered whether adding sema3A protein to the culture medium could also inhibit axon growth in the agomir‐treated DRG neurons. Sema3A evidently inhibited axon growth in agomir or scramble transfected DRG neurons compared with that in the agomir group (*P* < .05) (Figure 4C,D). These data indicated that sema3A could be a key molecule in the process of miR‐30b promoting primary sensory neuron axon growth.

### 3.5 miR‐30b inhibits the RhoA/ROCK pathway

**Figure 5:**
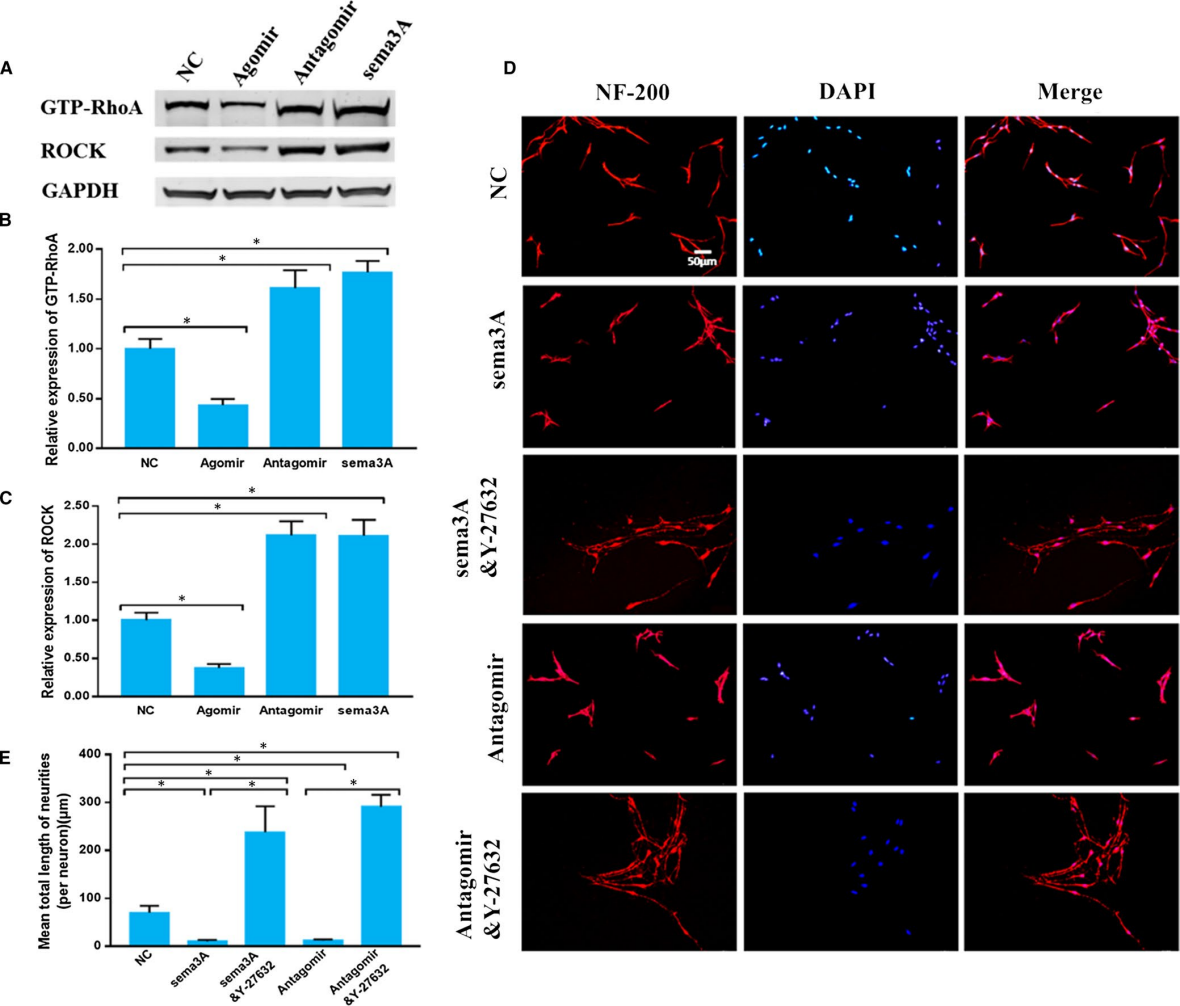
miR‐30b regulates the RhoA/ROCK pathway. A‐C, The expression of GTP‐RhoA and ROCK. D, Immunocytofluorescence images of cultured primary sensory neurons. Neurons are red, and cell nuclei are blue (200×). E, The mean total neurite length (per neuron). The data are expressed as the mean ± SD (one‐way ANOVA followed by Tukey's test; n = 4; *P < .05)

Although we have proven that miR‐30b could promote primary sensory neuron axon growth by targeting the sema3A/PlexinA1‐NRP‐1 axis, the associated intracellular pathway was still unknown. A previous study showed that the RhoA/ROCK pathway is the downstream pathway of sema3A in PC12 cells.^7^ However, whether RhoA/ROCK is the downstream pathway of miR‐30b in primary sensory neurons needed to be clarified. The expression of GTP‐RhoA and ROCK was up‐regulated in antagomir group and down‐regulated in agomir group compared with that in NC group (*P* < .05). Further sema3A treatment had an effect similar to that of the antagomir on the expression of GTP‐RhoA and ROCK (Figure 5A‐C). These data indicated that miR‐30b could regulate the sema3A/PlexinA1‐NRP‐1/RhoA/ROCK pathway in cultured primary sensory neurons.

However, whether RhoA/ROCK is the main downstream pathway in response to miR‐30b antagomir/sema3A stimulation in primary sensory neurons needed to be verified. We treated primary sensory neurons with antagomir/sema3A and the ROCK inhibitor Y‐27632. Antagomir and sema3A evidently decreased primary sensory neuron axon growth, while Y‐27632 greatly inhibited this decrease (*P* < .05) (Figure 5D,E). These data indicated that Y‐27632, the ROCK inhibitor, was sufficient to block the negative effect of miR‐30b antagomir/sema3A on axon growth in cultured primary sensory neurons. Thus, RhoA/ROCK was the key downstream pathway for sema3A to inhibit neurite growth.

### 3.6 miR‐30b promotes primary sensory neuron axon growth under NogoA‐FC inhibitory conditions

**Figure 6:**
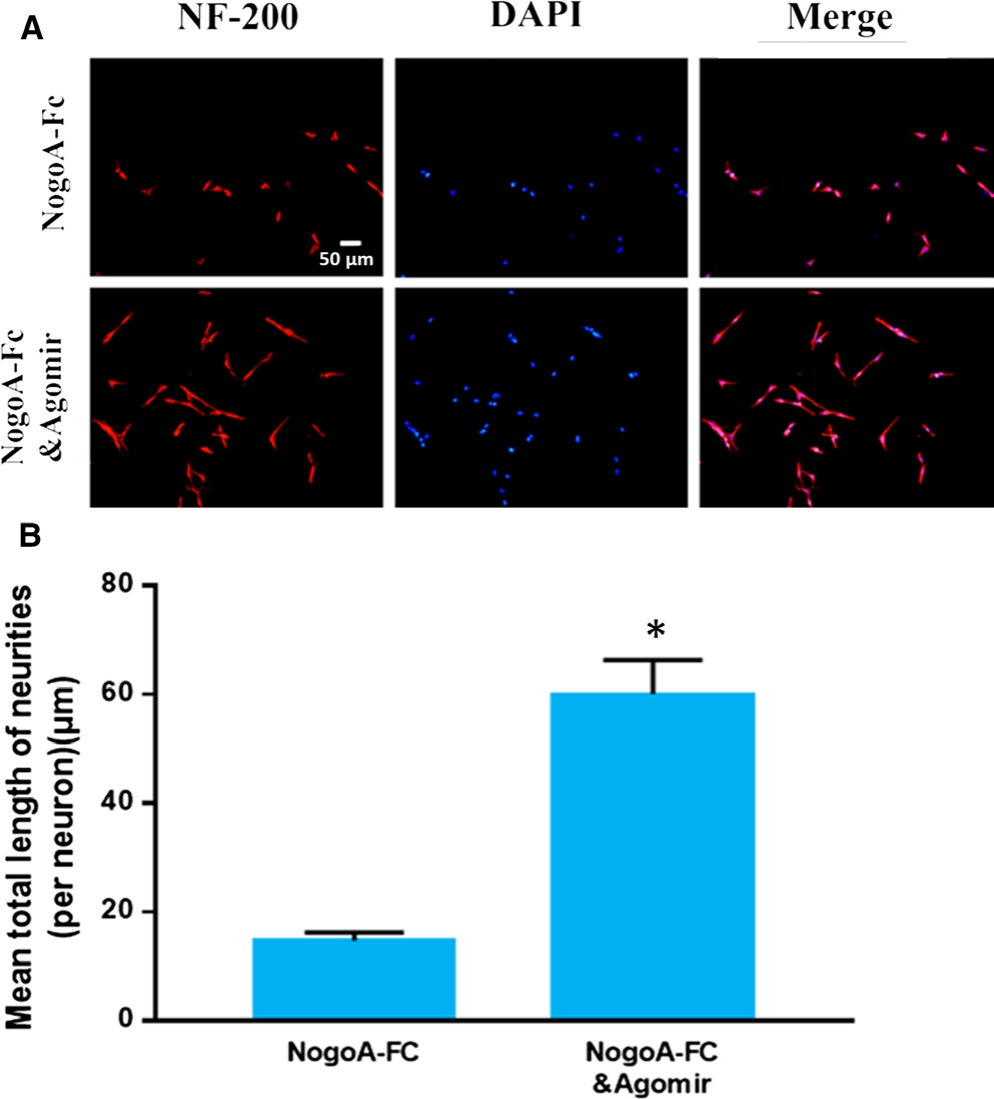
miR‐30b promotes primary sensory neuron axon growth under NogoA‐FC inhibitory conditions. A, Immunocytofluorescence images of cultured primary sensory neurons. Neurons are red, and cell nuclei are blue (200×). B, The mean total neurite length (per neuron). The data are expressed as the mean ± SD (one‐way ANOVA followed by Tukey's test; n = 4; *P < .05)

The RhoA/ROCK pathway is widely known to be associated with axon growth inhibition signalling in response to extracellular inhibitory molecules, such as NogoA, which binds to a membrane receptor.^30^ We proved that RhoA/ROCK was downstream of sema3A, which indicated that miR‐30b might facilitate primary sensory neuron axon growth in NogoA‐Fc inhibitory conditions by inhibiting the sema3A/NRP‐1/PlexinA1/RhoA/ROCK axis. We treated primary sensory neurons with NogoA‐Fc and miR‐30b agomir. In the NogoA‐Fc group, the axon length was inhibited, and the miR‐30b agomir promoted axon growth in NogoA‐Fc inhibitory conditions (Figure 6A,B). These data demonstrated that miR‐30b promoted primary sensory neuron axon growth in a NogoA‐Fc inhibitory microenvironment.

### 3.7 miR‐30b regulates the sema3A/PlexinA1‐NRP‐1/RhoA/ROCK pathway in vivo

**Figure 7:**
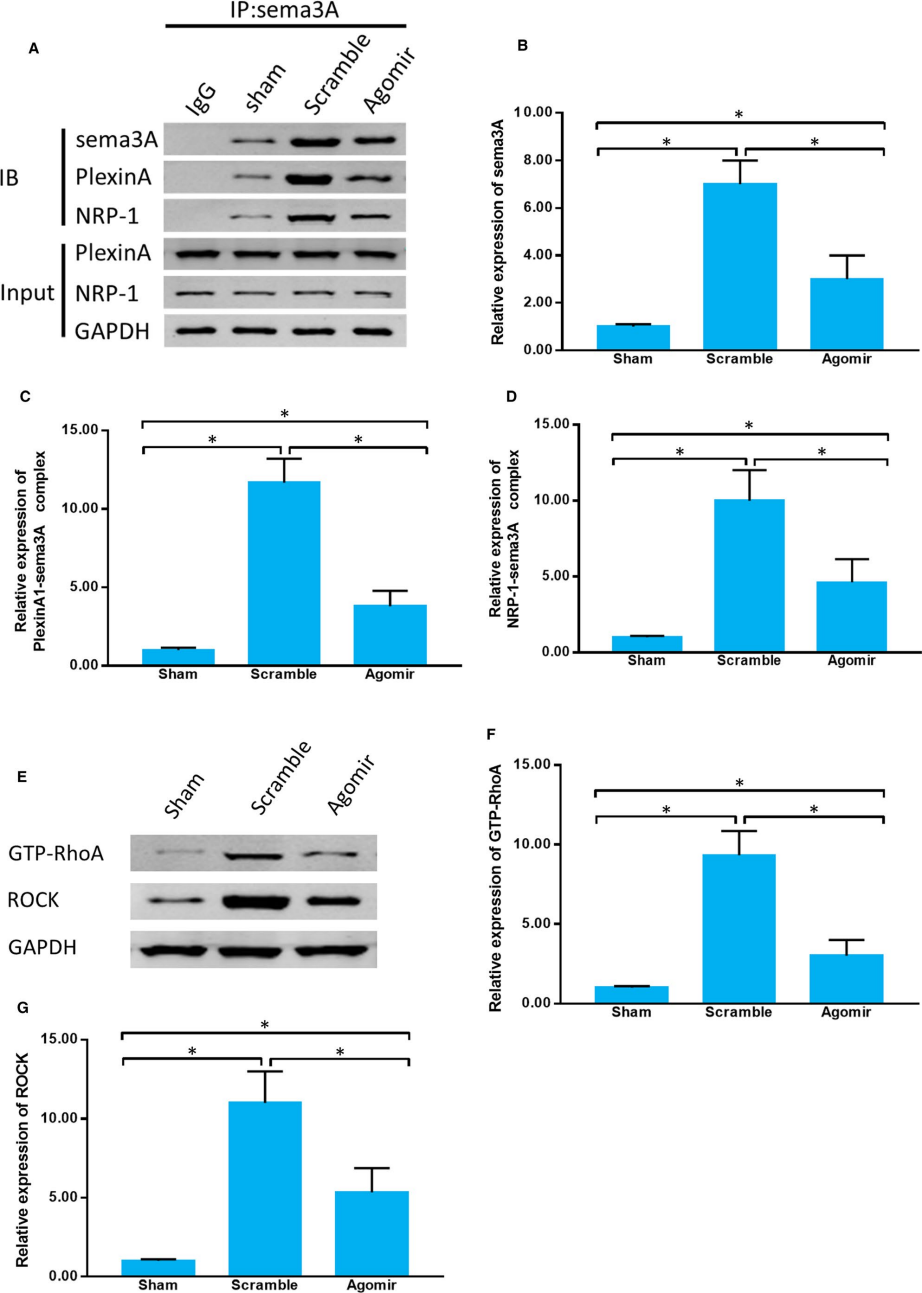
miR‐30b regulates the sema3A/PlexinA1‐NRP‐1/RhoA/ROCK pathway in vivo. A, The binding of sema3A, NRP‐1 and PlexinA1 was detected by coimmunoprecipitation assay. B‐D, Quantitative histogram showing the expression of sema3A, PlexinA1‐sema3A complex and NRP‐1‐sema3A complex. (E‐G) The expression of GTP‐RhoA and ROCK. Data are expressed as the mean ± SD of three experiments (n = 6, *P < .05)

To further clarify the regulation role of miR‐30b after SDCL, miR‐30b agomir was injected into the DRG tissues. We wondered whether agomir could also regulate sema3A/PlexinA1‐NRP‐1 complex formation and downstream RhoA/ROCK pathway. The sema3A expression was up‐regulated after SDCL and miR‐30b agomir reduced its expression. The formation of sema3A/PlexinA1 complex and sema3A/ NRP‐1 complex was induced in Scramble group compared with that in Sham group. The application of miR‐30b agomir reduced the formation of both complexes (Figure 7A‐D). The downstream proteins, GTP‐RhoA and ROCK, were also up‐regulated after SDCL, and miR‐30b agomir down‐regulated their expression (Figure 7E‐G). These results indicated miR‐30b agomir could also regulate the sema3A/PlexinA1‐NRP‐1/RhoA/ROCK pathway after SDCL.

### 3.8 miR‐30b promotes sensory conductive function recovery after SDCL

**Figure 8:**
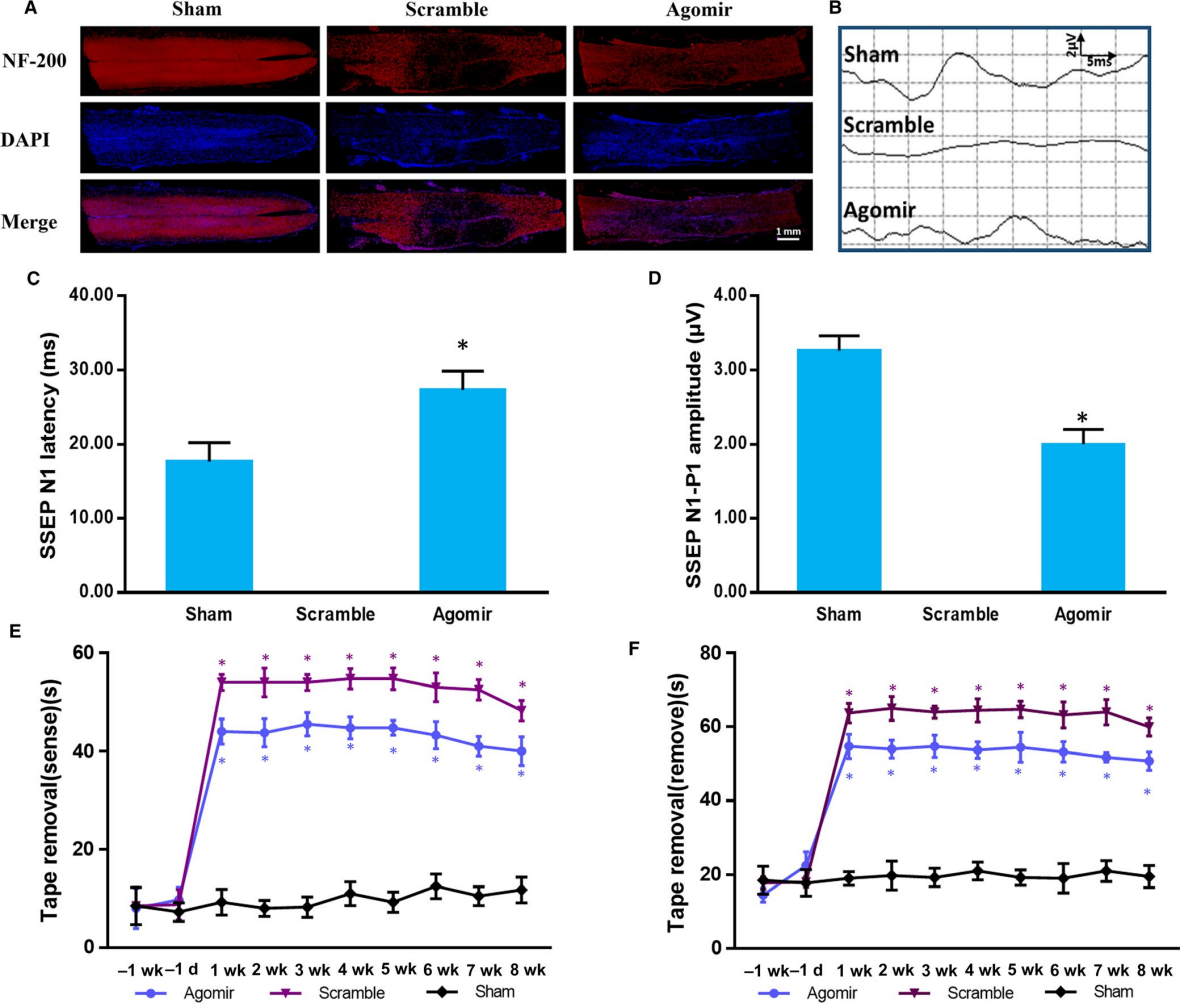
miR‐30b promotes sensory conductive function recovery after SDCL. A, NF‐200 Immunofluorescence staining of spinal cord dorsal column (B) Representative average waveform curves of SSEP examination. C, N latency quantitative histogram of SSEP. (D) N‐P amplitude quantitative histogram of SSEP. E, F, The latency of sensation and removal of the tape removal test. Data are expressed as the mean ± SD of three experiments (n = 6, *P < .05)

We wondered whether the application of miR‐30b agomir could promote spinal cord dorsal column axon regeneration and spinal cord sensory conductive function recovery after SDCL. The immunohistochemistry stain showed the NF‐200 positive area was larger in Agomir group compared with that in Scramble group, although the NF‐200 area in Agomir group was still smaller than that in Sham group (Figure 8A). The sensory conductive function was detected with SSEP and TRT. The SSEP results showed there was normal waveform in Sham group, while there was no recognized waveform in Scramble group. Excitingly, although the N peak latency was delayed and the N‐P amplitude was reduced compared with that in Sham group, there was detectable waveform in miR‐30b agomir group (Figure 8B‐D). The TRT showed the latencies of both sensing and removing of the tape were very short in Sham group rats. However, the latencies were delayed in both Scramble and Agomir group compared with that in Sham group (*P* < .05). Promisingly, compared with Scramble group, the latencies were reduced in Agomir group (*P* < .05) (Figure 8E,F). The results indicated miR‐30b agomir could promote spinal cord sensory conductive function recovery after SDCL.

## 4 DISCUSSION

SCI is a devastating disease with no effective treatment strategy in the clinic. Sensory dysfunction occurs immediately after SCI. Various sensory disorders, including chronic pain, central neuropathic pain and sensory paraesthesia, affect the patients’ life quality every second.^5^, ^31^ Central neuropathic pain especially tortures the patient due to the lack of an efficient treatment strategy, and the depression associated with central neuropathic pain could lead to suicide.^5^, ^32^ Thus, focusing on sensory neuron neurite regeneration to recover sensory function post‐spinal cord injury is particularly important. Different from the peripheral nerve system, injured axons in central nerve system show limited regeneration. The poor regeneration ability could be the results of decreased growth‐related genes activation.^33^ In addition, the inhibitory microenvironment that forms after injury is hostile to the regenerative process.^6^ Studies have focused on both promoting the intrinsic regenerative ability of neurons and neutralizing the inhibitory factors. The complex microenvironment consists of chemical repulsive molecules, such as sema3A, NogoA and TNF‐α, secreted by multiple cell types and physical glial scars that formed by astrocytes.^34^, ^35^ Further, although shown to have a positive effect, neutralizing inhibitory factors has a limited effect on axon regeneration.^6^, ^36^ Therefore, enhancing the intrinsic regenerative ability of neuronal axons in the central nervous system to conquer the negative microenvironment is of greater importance.

MiRNAs regulate at least 20%‐30% of gene expression at the translational level ^37^ and are widely studied in both the clinic ^38^ and in basic research.^16^, ^39^ In all types of neurons, miRNAs are widely expressed in cell body, axon and synapses and are involved in regulating multiple processes, such as development, differentiation, morphogenesis and plasticity,^40^, ^41^ suggesting that miRNAs could regulate intrinsic neuron regenerative capability. Previous studies have proven that miRNAs can regulate neuronal axon growth via different pathways.^15^, ^16^, ^42^ MiR‐30b could protect cell from MPP(+) treatment.^19^ Neuropathic pain could be relieved by miR‐30b overexpression.^20^, ^21^, ^22^ In this study, we wanted to clarify one of the mechanisms underlying axon growth regulation. miR‐30b has been proven to be expressed in DRG tissues to regulate pain by modulating Nav1.3 and Nav1.7.^22^, ^43^ Meanwhile, miR‐30b can also regulate retinal neuron axon growth.^23^ Because primary sensory neuron and retinal neuron have differences in structure and function, the interaction of miR‐30b and sema3A mRNA still needed to be verified in primary sensory neuron.

The related downstream pathway of miR‐30b in primary sensory neurons was also investigated. After a literature research and prediction using bioinformatics databases, miR‐30b was found to target sema3A and was chosen for further study. Sema3A is a neuron‐secreted chemical repulsive molecule that inhibits neuron axon growth in an autocrine manner.^8^ Different from other inhibitory molecules, including TNF‐α, IL‐1β, NogoA and MAG, which are secreted from multiple cell types, including astrocytes and oligodendrocytes,^44^, ^45^ the unique characteristics of sema3A indicate that it takes part in both the intrinsic regenerative ability and extrinsic microenvironment formation. After binding to its co‐receptor, NRP1 and PlexinA1, secreted sema3A immediately induces activation of RhoA. The expression of sema3A is paralleled with the expression of NRP‐1 and PlexinA1 in response to miR‐30b modulation. Inhibition of RhoA/ROCK with an inhibitor is sufficient to remove the new synthetic proteins required for sema3A‐induced growth cone collapse. Application of the ROCK inhibitor Y‐27632 facilitated neuronal axon growth under miR‐30b antagomir/sema3A conditions. The RhoA/ROCK pathway is the intracellular signalling response to extracellular inhibitory molecules, such as NogoA, MAG and CSPGs.^46^ We demonstrated that up‐regulation of miR‐30b was sufficient to inhibit sema3A expression and RhoA/ROCK pathway activation in vitro and in vivo. The up‐regulation of miR‐30b could promote primary sensory neuron neurite growth and spinal cord sensory conductive function recovery. SSEP and TRT are sensitive detections that could reflect the spinal cord sensory conductive function.^47^, ^48^

Overall, in this study, we identified miR‐30b as an important regulator of primary sensory neuron axon growth through inhibiting sema3A expression and downstream RhoA/ROCK pathway. To our knowledge, this was the first study clarifying the role of miR‐30b in spinal cord dorsal column injury. These results enriched the molecular mechanism of spinal cord dorsal column injury and provided a new treatment strategy and novel target for treating sensory conductive function dysfunction after spinal cord injury.

## CONFLICT OF INTEREST

All authors claim that there are no conflicts of interest.

## AUTHOR CONTRIBUTION

**Xin Wang:** Data curation (equal); Writing‐original draft (equal). **Bo Li:** Writing‐review & editing (lead). **Zhijie Wang:** Data curation (equal); Resources (equal); Visualization (equal); Writing‐review & editing (supporting). **Fengyan Wang:** Writing‐review & editing (lead). **Jing Liang:** Data curation (lead); Investigation (equal). **Chuanjie Chen:** Formal analysis (lead); Funding acquisition (equal). **Lei Zhao:** Methodology (lead). **Bo Zhou:** Software (lead). **Xiaoling Guo:** Conceptualization (equal); Validation (equal). **Liqun Ren:** Conceptualization (equal). **Xin Yuan:** Formal analysis (lead). **Xueming Chen:** Conceptualization (equal); Funding acquisition (equal). **Tianyi Wang:** Conceptualization (equal); Funding acquisition (equal); Supervision (equal).

## Data Availability

The data that support the findings of this study are available from the corresponding author upon reasonable request.
